# Usefulness of Vena Contracta for Identifying Severe Secondary Mitral Regurgitation: A Three-Dimensional Transesophageal Echocardiography Study

**DOI:** 10.31083/j.rcm2408233

**Published:** 2023-08-15

**Authors:** Hirokazu Onishi, Masaki Izumo, Toru Naganuma, Yoshihiro J. Akashi, Sunao Nakamura

**Affiliations:** ^1^Department of Cardiology, New Tokyo Hospital, 270-2232 Chiba, Japan; ^2^Department of Cardiology, St. Marianna University School of Medicine, 216-8511 Kanagawa, Japan

**Keywords:** secondary mitral regurgitation, vena contracta width, vena contracta area, effective regurgitant orifice area

## Abstract

**Background:**

In secondary mitral regurgitation (SMR), effective regurgitant orifice area by the proximal isovelocity surface area method (${\mathrm{EROA}}_{\text{PISA}}$) evaluation might cause an underestimation of regurgitant orifice area because of its ellipticity compared with vena contracta area (VCA). We aimed to reassess the SMR severity using VCA-related parameters and ${\mathrm{EROA}}_{\text{PISA}}$.

**Methods:**

The three-dimensional transesophageal echocardiography data of 128 patients with SMR were retrospectively analyzed; the following parameters were evaluated: ${\mathrm{EROA}}_{\text{PISA}}$, anteroposterior and mediolateral vena contracta widths (VCWs) of VCA (i.e., ${\mathrm{VCW}}_{\text{AP}}$ and ${\mathrm{VCW}}_{\text{ML}}$), ${\mathrm{VCW}}_{\text{Average}}$ calculated as (${\mathrm{VCW}}_{\text{AP}}$ + ${\mathrm{VCW}}_{\text{ML}}$)/2, and ${\mathrm{VCA}}_{\text{Ellipse}}$ calculated as π × (${\mathrm{VCW}}_{\text{AP}}$/2) × (${\mathrm{VCW}}_{\text{ML}}$/2). Severe SMR was defined as ≥0.39 ${\mathrm{cm}}^{2}$.

**Results:**

The mean age of the patients was 77.0 ± 8.9 years, and 78 (60.9%) were males. Compared with ${\mathrm{EROA}}_{\text{PISA}}$ (r = 0.801), ${\mathrm{VCW}}_{\text{Average}}$ (r = 0.940) and ${\mathrm{VCA}}_{\text{Ellipse}}$ (r = 0.980) were strongly correlated with VCA. On receiver-operating characteristic curve analysis, ${\mathrm{VCW}}_{\text{Average}}$ and ${\mathrm{VCA}}_{\text{Ellipse}}$ had C-statistics of 0.981 (95% confidence interval [CI], 0.963–1.000) and 0.985 (95% CI, 0.970–1.000), respectively; these were significantly higher than 0.910 (95% CI, 0.859–0.961) in ${\mathrm{EROA}}_{\text{PISA}}$ (*p* = 0.007 and *p* = 0.003, respectively). The best cutoff values for severe SMR of ${\mathrm{VCW}}_{\text{Average}}$ and ${\mathrm{VCA}}_{\text{Ellipse}}$ were 0.78 cm and 0.42 ${\mathrm{cm}}^{2}$, respectively. The prevalence of severe SMR significantly increased with an increase in ${\mathrm{EROA}}_{\text{PISA}}$ (38 of 88 [43.2%] patients with ${\mathrm{EROA}}_{\text{PISA}}$ <0.30 ${\mathrm{cm}}^{2}$, 21 of 24 [87.5%] patients with ${\mathrm{EROA}}_{\text{PISA}}$ = 0.30–0.40 ${\mathrm{cm}}^{2}$, and 16 of 16 [100%] patients with ${\mathrm{EROA}}_{\text{PISA}}$ ≥0.40 ${\mathrm{cm}}^{2}$ [Cochran–Armitage test; *p* < 0.001]). Among patients with ${\mathrm{EROA}}_{\text{PISA}}$ <0.30 ${\mathrm{cm}}^{2}$, SMR severity based on VCA was accurately reclassified using ${\mathrm{VCW}}_{\text{Average}}$ (McNemar’s test; *p* = 0.505) and ${\mathrm{VCA}}_{\text{Ellipse}}$ (*p* = 0.182).

**Conclusions:**

Among patients who had SMR with ${\mathrm{EROA}}_{\text{PISA}}$ of <0.30 ${\mathrm{cm}}^{2}$, suggestive of moderate or less SMR according to current guidelines, >40% had discordantly severe SMR based on VCA. ${\mathrm{VCW}}_{\text{Average}}$ and ${\mathrm{VCA}}_{\text{Ellipse}}$ values were useful for identifying severe SMR based on VCA in these patients.

## 1. Introduction

Secondary mitral regurgitation (SMR) is a common valvular heart disease that affects heart failure symptoms and clinical outcomes [1, 2, 3]. According to the current guidelines, two-dimensional (2D) echocardiographic parameters, including vena contracta width (VCW) and effective regurgitant orifice area by the proximal isovelocity surface area method (${\mathrm{EROA}}_{\text{PISA}}$), are recommended to determine SMR severity; however, the severity may be underestimated using VCW and ${\mathrm{EROA}}_{\text{PISA}}$ if regurgitant orifice area is elliptical [4, 5, 6].

Vena contracta area (VCA) hydrodynamically corresponds to the regurgitant orifice area [7]. Kahlert *et al*. [8] primarily reported direct planimetry of VCA (${\mathrm{VCA}}_{\text{3D}}$) based on three-dimensional transesophageal echocardiography (3D-TEE), and ${\mathrm{VCA}}_{\text{3D}}$ was subsequently validated using an *in vitro* model and cardiac magnetic resonance imaging [9, 10]. Furthermore, Goebel *et al*. [11] reported that compared with ${\mathrm{EROA}}_{\text{PISA}}$, ${\mathrm{VCA}}_{\text{3D}}$ is a robust parameter for discriminating severe SMR. Moreover, previous studies have suggested that ${\mathrm{VCA}}_{\text{3D}}$ is elliptical in cases of SMR based on several vena contracta (VC) parameters, including anteroposterior VCW (${\mathrm{VCW}}_{\text{AP}}$), mediolateral VCW (${\mathrm{VCW}}_{\text{ML}}$), average of ${\mathrm{VCW}}_{\text{AP}}$ and ${\mathrm{VCW}}_{\text{ML}}$ (${\mathrm{VCW}}_{\text{Average}}$), and VCA calculated as an ellipse (${\mathrm{VCA}}_{\text{Ellipse}}$). These studies have also reported that the ellipticity consequently limited the ability of ${\mathrm{VCW}}_{\text{AP}}$ and ${\mathrm{EROA}}_{\text{PISA}}$ to accurately classify SMR severity [8, 12]. However, these were relatively small-scale studies, and there is little information available regarding the best cutoff values of VC parameters for severe SMR.

Thus, we hypothesized that parameters that considered the elliptical shape of the mitral regurgitant orifice, including ${\mathrm{VCA}}_{\text{Average}}$ and ${\mathrm{VCA}}_{\text{Ellipse}}$, are better surrogate markers for severe SMR based on ${\mathrm{VCA}}_{\text{3D}}$ than ${\mathrm{EROA}}_{\text{PISA}}$. This study also investigated the best cutoff values of these VC parameters for severe SMR. Furthermore, we reassessed the true SMR severity using the cutoff values of VC parameters to avoid underestimating SMR based on ${\mathrm{EROA}}_{\text{PISA}}$.

## 2. Methods

### 2.1 Patient Population

Patient characteristics and echocardiographic data were collected from the medical records and echocardiography reports. The study protocol was approved by the Institutional Review Board of New Tokyo Hospital and was in accordance with the guidelines of the Declaration of Helsinki. The requirement for informed consent was waived because of the retrospective nature of this study. Based on integrative methods using qualitative, semiquantitative, and quantitative approaches, 154 patients with at least mild SMR were identified via a review of echocardiography databases at New Tokyo Hospital between January 2018 and March 2021. These patients underwent 3D-TEE based on clinical indications and transthoracic echocardiography (TTE) within 1 month of 3D-TEE at our center [4]. SMR was defined as incomplete mitral leaflet closure because of regional myocardial dysfunction, global left ventricular remodeling, apical tethering of the mitral valve (MV), or annular dilation in the presence of an anatomically normal valve apparatus [4, 13]. Of 172 patients, those with multiple or nonholosystolic SMR jet (6 patients), previous MV intervention (7 patients), concomitant mitral stenosis (2 patients) [14], and mitral annular calcification (3 patients) were excluded from this study.

Overall, 19 of 154 patients were excluded because the quality of 3D imaging was inadequate for ${\mathrm{VCA}}_{\text{3D}}$ analysis, and 7 patients were excluded because of incomplete data for the quantitative assessment of SMR; hence, 128 patients were included in the final analysis.

### 2.2 Echocardiographic Parameters

Echocardiographic examinations were performed using iE33 system (Philips Healthcare, Andover, MA, USA) and EPIQ7 system (Philips Healthcare, Andover, MA, USA) equipped with a matrix-array transducer for transthoracic (X5-1) and transesophageal echocardiography (X7-2t and X8-2t), according to the guidelines for the clinical application of echocardiography [4, 14, 15, 16, 17, 18]. For offline analysis, echocardiographic data were stored in a computer at a dedicated workstation.

Regarding two-dimensional TTE (2D-TTE) parameters, left ventricular end-diastolic and -systolic volumes, left ventricular ejection fraction (LVEF), and left atrial volume were estimated using the biplane Simpson disk method via transthoracic echocardiography.

**Fig. 1.**
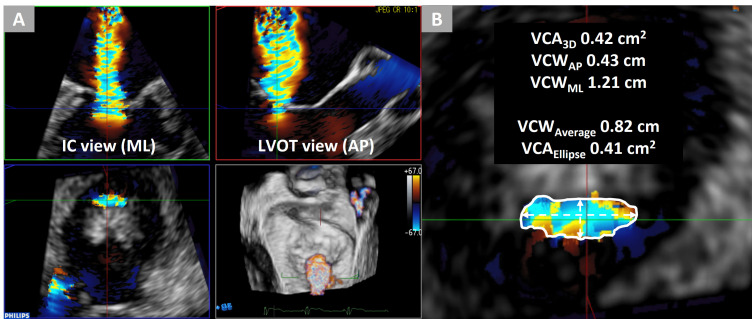
**Assessment of vena contracta using 3D-TEE**. A case of an 84-year-old woman with dilated cardiomyopathy and secondary mitral regurgitation. (A) Vena contracta described by multiplanar reconstruction of 3D color Doppler datasets. (B) ${\mathrm{VCA}}_{\text{3D}}$ measured using manual planimetry of the vena contracta was 0.42 ${\mathrm{cm}}^{2}$. ${\mathrm{VCW}}_{\text{AP}}$ and ${\mathrm{VCA}}_{\text{ML}}$ measured as the narrow and wide VCWs in the anteroposterior and mediolateral directions were 0.43 and 1.21 cm, respectively. ${\mathrm{VCW}}_{\text{Average}}$, calculated as (${\mathrm{VCW}}_{\text{AP}}$ + ${\mathrm{VCW}}_{\text{ML}}$)/2, was 0.82 cm. ${\mathrm{VCA}}_{\text{Ellipse}}$, calculated as π × (${\mathrm{VCW}}_{\text{AP}}$/2) × (${\mathrm{VCW}}_{\text{ML}}$/2), was 0.41 ${\mathrm{cm}}^{2}$. IC, intercommissural; LVOT, left ventricular outflow tract; 3D-TEE, three-dimensional transesophageal echocardiography; ${\mathrm{VCA}}_{\text{3D}}$, three-dimensional vena contracta area; ${\mathrm{VCW}}_{\text{AP}}$, anteroposterior vena contracta width; ${\mathrm{VCW}}_{\text{ML}}$, mediolateral vena contracta width; ${\mathrm{VCW}}_{\text{Average}}$, average of anteroposterior and mediolateral vena contracta widths; ${\mathrm{VCA}}_{\text{Ellipse}}$, vena contracta area as an ellipse.

Regarding TEE parameters, ${\mathrm{EROA}}_{\text{PISA}}$ and regurgitant volume (${\mathrm{RV}}_{\text{PISA}}$) were estimated using the proximal isovelocity surface area method [4]. A continuous wave Doppler cursor was aligned parallel to the SMR jet for obtaining peak velocity and velocity–time integral at a Nyquist limit of 50–70 cm/s, with the gain set to a level immediately below the threshold for noise. ${\mathrm{EROA}}_{\text{PISA}}$ was derived using a color Doppler in a four-chamber view at an aliasing velocity of 30–40 cm/s. Moreover, during systole, proximal isovelocity surface area (PISA) radius and flow velocity parameters were obtained at similar time points for calculating ${\mathrm{EROA}}_{\text{PISA}}$. To determine VC parameters, 3D color Doppler datasets were acquired from an intercommissural view using full volume for each patient. The quantification of ${\mathrm{VCA}}_{\text{3D}}$ was performed via multiplanar reconstruction using dedicated software (Philips QLAB Versions 9.0, Philips Healthcare, Andover, MA, USA) (Fig. 1) [4]. The cropping plane was moved along the direction of the jet until the smallest jet cross-sectional area became visible at the level of VC. Subsequently, ${\mathrm{VCA}}_{\text{3D}}$ was measured using manual planimetry of the color Doppler flow signal. ${\mathrm{VCW}}_{\text{AP}}$ and ${\mathrm{VCW}}_{\text{ML}}$ were also measured as anteroposterior and mediolateral VCWs, respectively, in reconstructed 2D planes from the 3D-TEE dataset; ${\mathrm{VCW}}_{\text{AP}}$ and ${\mathrm{VCW}}_{\text{ML}}$ were obtained in the left ventricular outflow tract and intercommissural views (or views that were close to intercommissural views), respectively [8]. ${\mathrm{VCW}}_{\text{Average}}$ was calculated as (${\mathrm{VCW}}_{\text{AP}}$ + ${\mathrm{VCW}}_{\text{ML}}$)/2, ${\mathrm{VCA}}_{\text{Ellipse}}$ was calculated as π × (${\mathrm{VCW}}_{\text{AP}}$/2) × (${\mathrm{VCW}}_{\text{ML}}$/2) [8], and ${\mathrm{VCA}}_{\text{3D}}$ shape index was calculated as ${\mathrm{VCW}}_{\text{ML}}$/${\mathrm{VCW}}_{\text{AP}}$. In patients with irregular rhythm (i.e., atrial fibrillation or flutter not requiring constant ventricular pacing for bradycardia), these parameters were calculated as the mean of 3–5 parameters performed by avoiding remarkable irregular RR intervals. ${\mathrm{EROA}}_{\text{PISA}}$ and VC parameters were performed by one observer (H.O.).

${\mathrm{VCA}}_{\text{3D}}$ of ≥0.39 ${\mathrm{cm}}^{2}$ was used as a reference standard of severe SMR in the current study, considering that the severity of SMR may be underestimated using ${\mathrm{EROA}}_{\text{PISA}}$ and that ${\mathrm{VCA}}_{\text{3D}}$ is a more robust parameter for distinguishing severe SMR than ${\mathrm{EROA}}_{\text{PISA}}$ [4, 11].

### 2.3 Statistical Analysis

Categorical variables were presented as frequencies and analyzed using chi-square, Fisher’s exact, or Cochran–Armitage test, as appropriate. Continuous variables were presented as mean ± standard deviation or median with interquartile range and were compared using Mann–Whitney U or Jonckheere–Terpstra test, as appropriate. The overall rates of correct SMR severity classifications based on ${\mathrm{VCA}}_{\text{3D}}$ were statistically compared using McNemar’s test in 2 × 2 tables. Correlations between different parameters were determined using Pearson’s test and linear regression analysis. Receiver operating characteristic (ROC) curve analyses were performed to assess the ability of each parameter to identify severe SMR based on ${\mathrm{VCA}}_{\text{3D}}$. The Youden index was used to determine the best cutoff value for severe SMR based on ${\mathrm{VCA}}_{\text{3D}}$ considering optimal sensitivity and specificity. Discrimination of severe SMR based on ${\mathrm{VCA}}_{\text{3D}}$ was assessed using the C-statistic. All statistical tests were two-tailed, and a two-sided *p*-value of <0.05 was considered to indicate statistical significance. Data analysis was performed using EZR software version 1.50 (Saitama Medical Center, Jichi Medical University, Saitama, Japan) [19].

## 3. Results

### 3.1 Patient Characteristics

The mean age of the patients was 77.0 ± 8.9 years, and 78 (60.9%) patients were men (Table 1). Regarding echocardiographic data, the mean LVEF was 37.5% ± 13.4%, with an LVEF of <50% in 95 (74.2%) patients (Table 2). The mean tenting height of MV was 0.88 ± 0.34 cm. Regarding SMR quantification, ${\mathrm{EROA}}_{\text{PISA}}$ and ${\mathrm{RV}}_{\text{PISA}}$ were 0.26 ± 0.12 ${\mathrm{cm}}^{2}$ and 40.6 ± 17.3 mL, respectively, with severe SMR based on ${\mathrm{EROA}}_{\text{PISA}}$ of ≥0.40 ${\mathrm{cm}}^{2}$ (according to the current guidelines) in 16 (12.5%) patients [4]. ${\mathrm{VCA}}_{\text{3D}}$ was 0.46 ± 0.26 ${\mathrm{cm}}^{2}$, with severe SMR based on ${\mathrm{VCA}}_{\text{3D}}$ in 75 (58.6%) patients. ${\mathrm{VCW}}_{\text{Average}}$ and ${\mathrm{VCA}}_{\text{Ellipse}}$ were 0.84 ± 0.26 cm and 0.49 ± 0.28 ${\mathrm{cm}}^{2}$, respectively.

**Table 1.** **Patient demographics**.

Variables		All patients (n = 128)
Age, years		77.0 ± 8.9
Men, n		78 (60.9)
Body surface area, $m^{2}$		1.57 ± 0.17
Hypertension, n		66 (51.6)
Diabetes mellitus, n		42 (32.8)
Dyslipidemia, n		59 (46.1)
Smoking, n		71 (55.5)
Chronic kidney disease (eGFR <60 mL/min/1.73 $m^{2}$), n		108 (84.4)
Paroxysmal atrial fibrillation/flutter, n		32 (25.0)
Persistent atrial fibrillation/flutter, n		64 (50.0)
Irregular rhythm, n		54 (42.2)
Previous myocardial infarction, n		35 (27.3)
Pacemaker, n		16 (12.5)
Implantable cardioverter defibrillator, n		16 (12.5)
Cardiac resynchronization therapy, n		7 (5.5)
NYHA functional class		2.1 ± 0.6
	I, n	19 (14.8)
	II, n	85 (66.4)
	III, n	23 (18.0)
	IV, n	1 (0.8)

Continuous data are presented as means ± standard deviations, except brain natriuretic peptide (median and interquartile range); categorical data are given as the counts (percentages). eGFR, estimated glomerular filtration rate; NYHA, New York Heart Association.

**Table 2.** **Echocardiographic data**.

Variables		All patients (n = 128)
Measurements on two-dimensional transthoracic echocardiography		
	LVEDV index, mL/$m^{2}$	120.6 ± 50.0
	LVESV index, mL/$m^{2}$	83.9 ± 47.9
	LVEF, %	37.5 ± 13.4
	LVEF <50%, n	95 (74.2)
	Interventricular septum thickness, mm	9.3 ± 1.9
	Posterior wall thickness, mm	9.2 ± 1.8
	Left atrial volume index, mL/$m^{2}$	119.8 ± 72.6
	PASP, mmHg	41.6 ± 14.0
	Severe aortic stenosis, n	0 (0.0)
	Severe aortic regurgitation, n	3 (2.3)
	Severe mitral stenosis, n	0 (0.0)
	Severe tricuspid regurgitation, n	32 (25.0)
	Severe pulmonary regurgitation, n	0 (0.0)
	Atrial septal defect, n	5 (3.9)
Measurements in mitral valve on three-dimensional transesophageal echocardiography		
	Heart rate, bpm	70.0 ± 10.3
	Heart rate in 54 patients with irregular rhythm, bpm	71.9 ± 10.4
	Anterior mitral leaflet pseudoprolapse, n	42 (33.0)
	Tenting height, cm	0.88 ± 0.34
	Anteroposterior annulus diameter, cm	3.28 ± 0.43
	Mediolateral annulus diameter, cm	3.49 ± 0.43
	${\mathrm{EROA}}_{\text{PISA}}$, ${\mathrm{cm}}^{2}$	0.26 ± 0.12
	${\mathrm{RV}}_{\text{PISA}}$, mL	40.6 ± 17.3
	Severe SMR based on ${\mathrm{EROA}}_{\text{PISA}}$ of ≥0.40 ${\mathrm{cm}}^{2}$, n	16 (12.5)
	${\mathrm{VCW}}_{\text{AP}}$, cm	0.49 ± 0.14
	${\mathrm{VCW}}_{\text{ML}}$, cm	1.19 ± 0.44
	${\mathrm{VCA}}_{\text{3D}}$, ${\mathrm{cm}}^{2}$	0.46 ± 0.26
	Severe SMR based on ${\mathrm{VCA}}_{\text{3D}}$ of ≥0.39 ${\mathrm{cm}}^{2}$, n	75 (58.6)
	${\mathrm{VCW}}_{\text{Average}}$, cm	0.84 ± 0.26
	Severe SMR based on ${\mathrm{VCA}}_{\text{Average}}$ of ≥0.78 cm, n	72 (56.3)
	${\mathrm{VCA}}_{\text{Ellipse}}$, ${\mathrm{cm}}^{2}$	0.49 ± 0.28
	Severe SMR based on ${\mathrm{VCA}}_{\text{Ellipse}}$ of ≥0.42 ${\mathrm{cm}}^{2}$, n	70 (54.7)
	${\mathrm{VCA}}_{\text{3D}}$ shape index	2.47 ± 0.84
	Frame rate in ${\mathrm{VCA}}_{\text{3D}}$ measurements, Hz	18.4 ± 6.1
	Frame rate in ${\mathrm{VCA}}_{\text{3D}}$ measurements in 54 patients with irregular rhythm, Hz	18.8 ± 5.4

Continuous data are presented as means ± standard deviations; categorical data are given as the counts (percentages). LVEDV, left ventricular end-diastolic volume; LVESV, left ventricular end-systolic volume; LVEF, left ventricular ejection fraction; PASP, pulmonary artery systolic pressure; ${\mathrm{EROA}}_{\text{PISA}}$, effective regurgitant orifice area by the proximal isovelocity surface area method; ${\mathrm{RV}}_{\text{PISA}}$, regurgitant volume based on proximal isovelocity surface area method; SMR, secondary mitral regurgitation; ${\mathrm{VCW}}_{\text{AP}}$, anteroposterior vena contracta width; ${\mathrm{VCW}}_{\text{ML}}$, mediolateral vena contracta width; ${\mathrm{VCA}}_{\text{3D}}$, vena contracta area based on three-dimensional echocardiographic data; ${\mathrm{VCW}}_{\text{Average}}$, averaged vena contracta width; ${\mathrm{VCA}}_{\text{Ellipse}}$, elliptical vena contracta area.

### 3.2 Associations of ${\mathrm{EROA}}_{\text{PISA}}$ with ${\mathrm{VCA}}_{\text{3D}}$

**Fig. 2.**
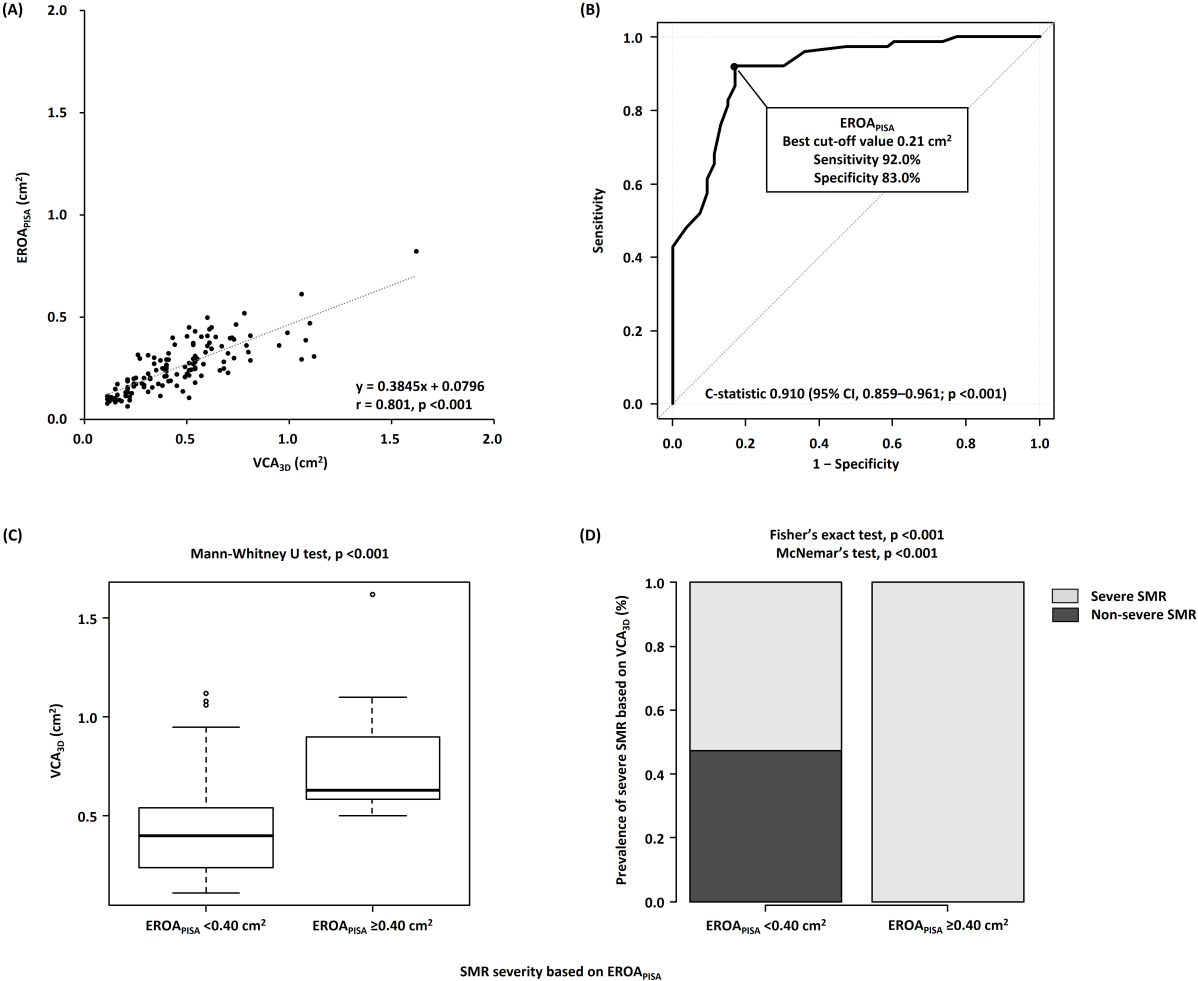
**Associations of ${\mathrm{VCA}}_{\text{𝟑𝐃}}$ with ${\mathrm{EROA}}_{\text{𝐏𝐈𝐒𝐀}}$**. (A) Correlations between ${\mathrm{VCA}}_{\text{3D}}$ and ${\mathrm{EROA}}_{\text{PISA}}$. (B) Receiver operating characteristic curve analyses of ${\mathrm{EROA}}_{\text{PISA}}$ to identify severe SMR. (C) Comparison of ${\mathrm{VCA}}_{\text{3D}}$ between the nonsevere (${\mathrm{EROA}}_{\text{PISA}}$ of <0.40 ${\mathrm{cm}}^{2}$) and severe (${\mathrm{EROA}}_{\text{PISA}}$ of ≥0.40 ${\mathrm{cm}}^{2}$) SMR groups. (D) Incidence of severe SMR based on ${\mathrm{VCA}}_{\text{3D}}$ of ≥0.39 ${\mathrm{cm}}^{2}$ in the nonsevere (${\mathrm{EROA}}_{\text{PISA}}$ of <0.40 ${\mathrm{cm}}^{2}$) and severe (${\mathrm{EROA}}_{\text{PISA}}$ of ≥0.40 ${\mathrm{cm}}^{2}$) SMR groups. ${\mathrm{VCA}}_{\text{3D}}$, three-dimensional vena contracta area; ${\mathrm{EROA}}_{\text{PISA}}$, effective regurgitant orifice area by proximal isovelocity surface area method; SMR, secondary mitral regurgitation.

${\mathrm{EROA}}_{\text{PISA}}$ showed a strong correlation with ${\mathrm{VCA}}_{\text{3D}}$ (r = 0.801, *p* < 0.001) (Fig. 2A). ROC curve analysis revealed that ${\mathrm{EROA}}_{\text{PISA}}$ showed good discrimination of severe SMR based on ${\mathrm{VCA}}_{\text{3D}}$ (C-statistic, 0.910; 95% confidence interval [CI], 0.859–0.961; *p* < 0.001), with the best cutoff value of 0.21 ${\mathrm{cm}}^{2}$ (Fig. 2B). The sensitivity and specificity of ${\mathrm{EROA}}_{\text{PISA}}$ for severe SMR based on ${\mathrm{VCA}}_{\text{3D}}$ were as follows: ${\mathrm{EROA}}_{\text{PISA}}$ of 0.20 ${\mathrm{cm}}^{2}$, 92.0% and 73.6%; ${\mathrm{EROA}}_{\text{PISA}}$ of 0.30 ${\mathrm{cm}}^{2}$, 49.3% and 94.3%; and ${\mathrm{EROA}}_{\text{PISA}}$ of 0.40 ${\mathrm{cm}}^{2}$, 22.6% and 100.0%; respectively. In addition, ${\mathrm{VCA}}_{\text{3D}}$ and SMR incidence were significantly lower (*p* < 0.001) in patients with nonsevere SMR based on ${\mathrm{EROA}}_{\text{PISA}}$ of <0.40 ${\mathrm{cm}}^{2}$ (according to the current guidelines) than in those with severe SMR based on ${\mathrm{EROA}}_{\text{PISA}}$ of ≥0.40 ${\mathrm{cm}}^{2}$ (Fig. 2C,D) [4]. Notably, among 112 patients with nonsevere SMR based on ${\mathrm{EROA}}_{\text{PISA}}$ of <0.40 ${\mathrm{cm}}^{2}$, 59 (52.7%) had discordantly severe SMR based on ${\mathrm{VCA}}_{\text{3D}}$. SMR severity based on ${\mathrm{VCA}}_{\text{3D}}$ was not correctly reclassified as severe SMR by ${\mathrm{EROA}}_{\text{PISA}}$ (McNemar’s test; *p* < 0.001).

### 3.3 Associations of ${\mathrm{VCW}}_{\text{AP}}$ with ${\mathrm{VCA}}_{\text{3D}}$

${\mathrm{VCW}}_{\text{AP}}$ showed a strong correlation with ${\mathrm{VCA}}_{\text{3D}}$ (r = 0.786, *p* < 0.001). ROC curve analysis indicated that ${\mathrm{VCW}}_{\text{AP}}$ showed relatively good discrimination of severe SMR based on ${\mathrm{VCA}}_{\text{3D}}$ (C-statistic, 0.874; 95% CI, 0.812–0.936; *p* < 0.001), with the best cutoff value of 0.43 cm.

### 3.4 Associations of ${\mathrm{VCW}}_{\text{Average}}$ and ${\mathrm{VCA}}_{\text{Ellipse}}$ with ${\mathrm{VCA}}_{\text{3D}}$

**Fig. 3.**
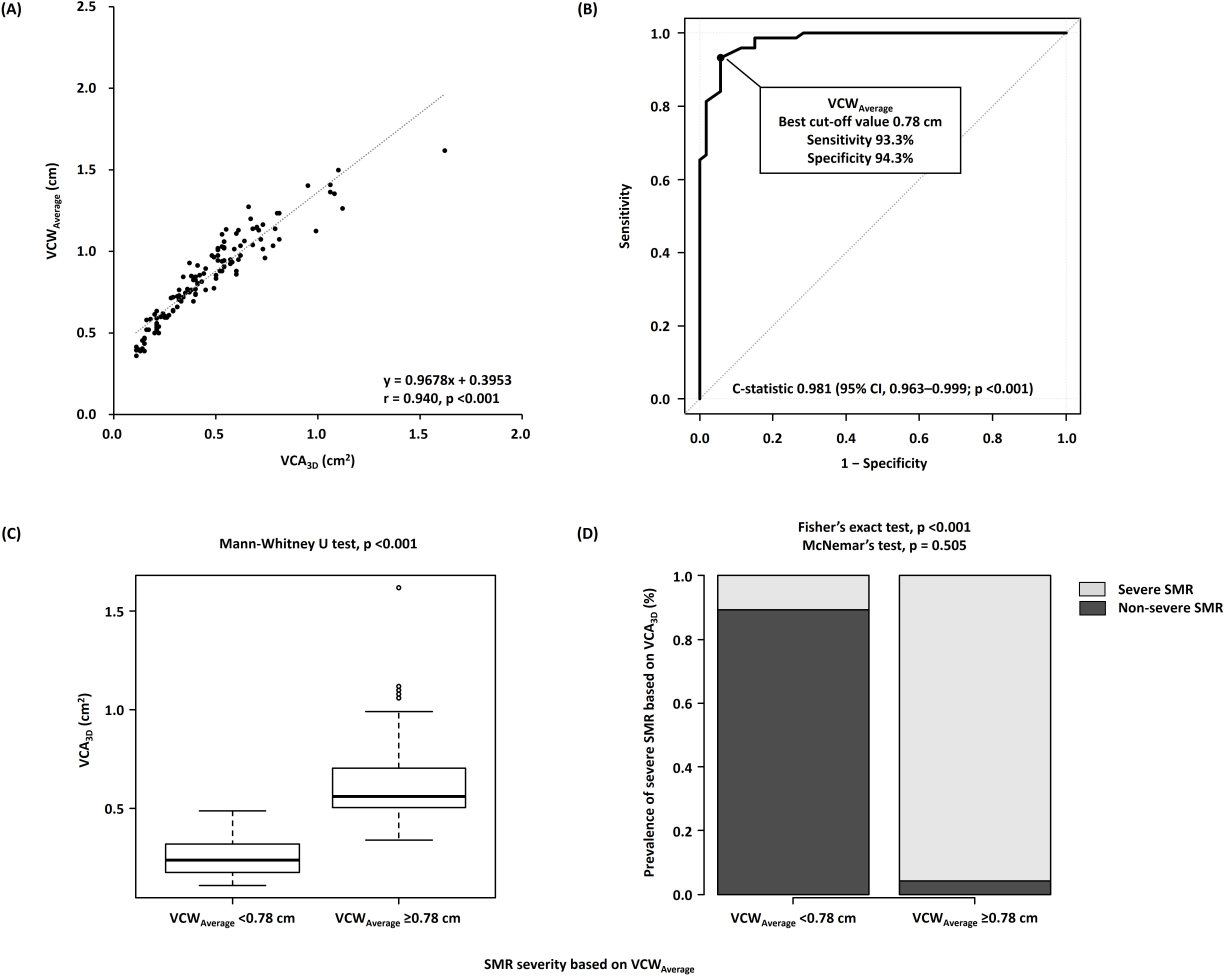
**Associations of ${\mathrm{VCA}}_{\text{𝟑𝐃}}$ with VCW${\mathrm{VCW}}_{\text{𝐀𝐯𝐞𝐫𝐚𝐠𝐞}}$**. (A) Correlations between ${\mathrm{VCA}}_{\text{3D}}$ and ${\mathrm{VCW}}_{\text{Average}}$. (B) Receiver operating characteristic curve analyses of ${\mathrm{VCW}}_{\text{Average}}$ to identify severe SMR. (C) Comparison of ${\mathrm{VCA}}_{\text{3D}}$ between the nonsevere (${\mathrm{VCW}}_{\text{Average}}$ of <0.78 cm) and severe (${\mathrm{VCW}}_{\text{Average}}$ of ≥0.78 cm) SMR groups. (D) Incidence of severe SMR based on ${\mathrm{VCA}}_{\text{3D}}$ of ≥0.39 ${\mathrm{cm}}^{2}$ in the nonsevere (${\mathrm{VCW}}_{\text{Average}}$ of <0.78 cm) and severe (${\mathrm{VCW}}_{\text{Average}}$ of ≥0.78 cm) SMR groups. ${\mathrm{VCA}}_{\text{3D}}$, three-dimensional vena contracta area; ${\mathrm{VCW}}_{\text{Average}}$, average of anteroposterior and mediolateral vena contracta widths; SMR, secondary mitral regurgitation.

**Fig. 4.**
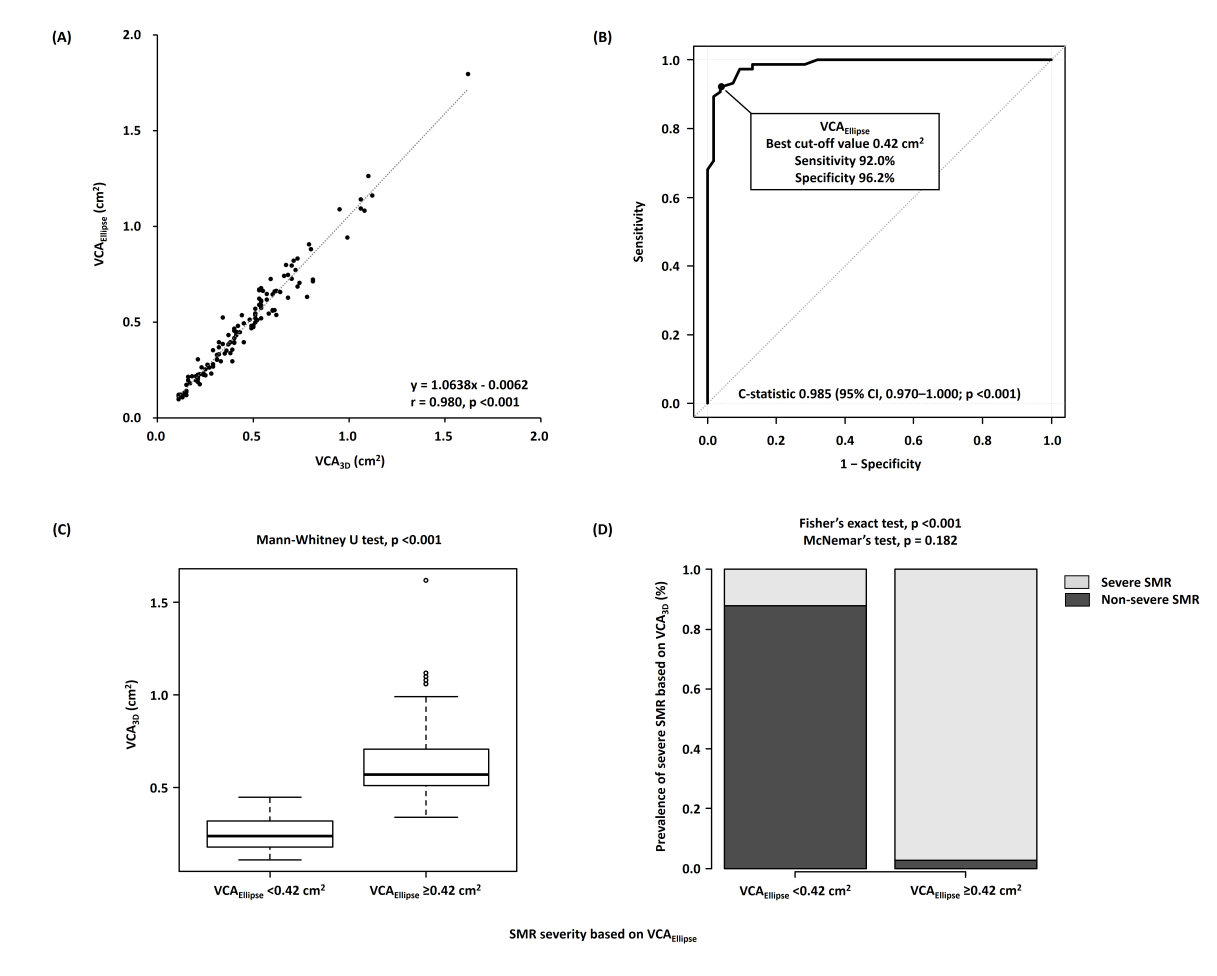
**Associations of ${\mathrm{VCA}}_{\text{𝟑𝐃}}$ with ${\mathrm{VCA}}_{\text{𝐄𝐥𝐥𝐢𝐩𝐬𝐞}}$**. (A) Correlations between ${\mathrm{VCA}}_{\text{3D}}$ and ${\mathrm{VCA}}_{\text{Ellipse}}$. (B) Receiver operating characteristic curve analyses of ${\mathrm{VCA}}_{\text{Ellipse}}$ to identify severe SMR. (C) Comparison of ${\mathrm{VCA}}_{\text{3D}}$ between the nonsevere (${\mathrm{VCA}}_{\text{Ellipse}}$ of <0.42 ${\mathrm{cm}}^{2}$) and severe (${\mathrm{VCA}}_{\text{Ellipse}}$ of ≥0.42 ${\mathrm{cm}}^{2}$) SMR groups. (D) Incidence of severe SMR based on ${\mathrm{VCA}}_{\text{3D}}$ of ≥0.39 ${\mathrm{cm}}^{2}$ in the nonsevere (${\mathrm{VCA}}_{\text{Ellipse}}$ of <0.42 ${\mathrm{cm}}^{2}$) and severe (${\mathrm{VCA}}_{\text{Ellipse}}$ of ≥0.42 ${\mathrm{cm}}^{2}$) SMR groups. ${\mathrm{VCA}}_{\text{3D}}$, three-dimensional vena contracta area; ${\mathrm{VCA}}_{\text{Ellipse}}$, vena contracta area as an ellipse; SMR, secondary mitral regurgitation.

${\mathrm{VCW}}_{\text{Average}}$ and ${\mathrm{VCA}}_{\text{Ellipse}}$ had a strong correlation with ${\mathrm{VCA}}_{\text{3D}}$ (r = 0.940, *p* < 0.001 and r = 0.980, *p* < 0.001, respectively) (Figs. 3A,4A). According to ROC curve analysis, ${\mathrm{VCW}}_{\text{Average}}$ and ${\mathrm{VCA}}_{\text{Ellipse}}$ showed fairly good discrimination of severe SMR based on ${\mathrm{VCA}}_{\text{3D}}$ (C-statistic, 0.981; 95% CI, 0.963–1.000; *p* < 0.001 and C-statistic, 0.985; 95% CI, 0.970–1.000; *p* < 0.001, respectively), with the best cutoff values of 0.78 cm and 0.42 ${\mathrm{cm}}^{2}$, respectively (Figs. 3B,4B). Moreover, regarding the comparison of C-statistics, ${\mathrm{VCW}}_{\text{Average}}$ and ${\mathrm{VCA}}_{\text{Ellipse}}$ showed significantly better discrimination than ${\mathrm{EROA}}_{\text{PISA}}$ (*p* = 0.007 and *p* = 0.003, respectively).

In addition, patients with nonsevere SMR, according to ${\mathrm{VCW}}_{\text{Average}}$ of <0.78 cm and ${\mathrm{VCA}}_{\text{Ellipse}}$ of <0.42 ${\mathrm{cm}}^{2}$, showed significantly lower ${\mathrm{VCA}}_{\text{3D}}$ (*p* < 0.001 for both) and SMR incidence based on ${\mathrm{VCA}}_{\text{3D}}$ (*p* < 0.001 for both) than those with severe SMR based on ${\mathrm{VCW}}_{\text{Average}}$ and ${\mathrm{VCA}}_{\text{Ellipse}}$ (Fig. 3C,D and Fig. 4C,D). Notably, SMR severity based on ${\mathrm{VCA}}_{\text{3D}}$ was correctly reclassified as severe SMR based on ${\mathrm{VCW}}_{\text{Average}}$ (*p* = 0.505) and ${\mathrm{VCA}}_{\text{Ellipse}}$ (*p* = 0.182).

### 3.5 SMR Severity Based on ${\mathrm{EROA}}_{\text{PISA}}$ Considering ${\mathrm{VCW}}_{\text{Average}}$ and ${\mathrm{VCA}}_{\text{Ellipse}}$

**Fig. 5.**
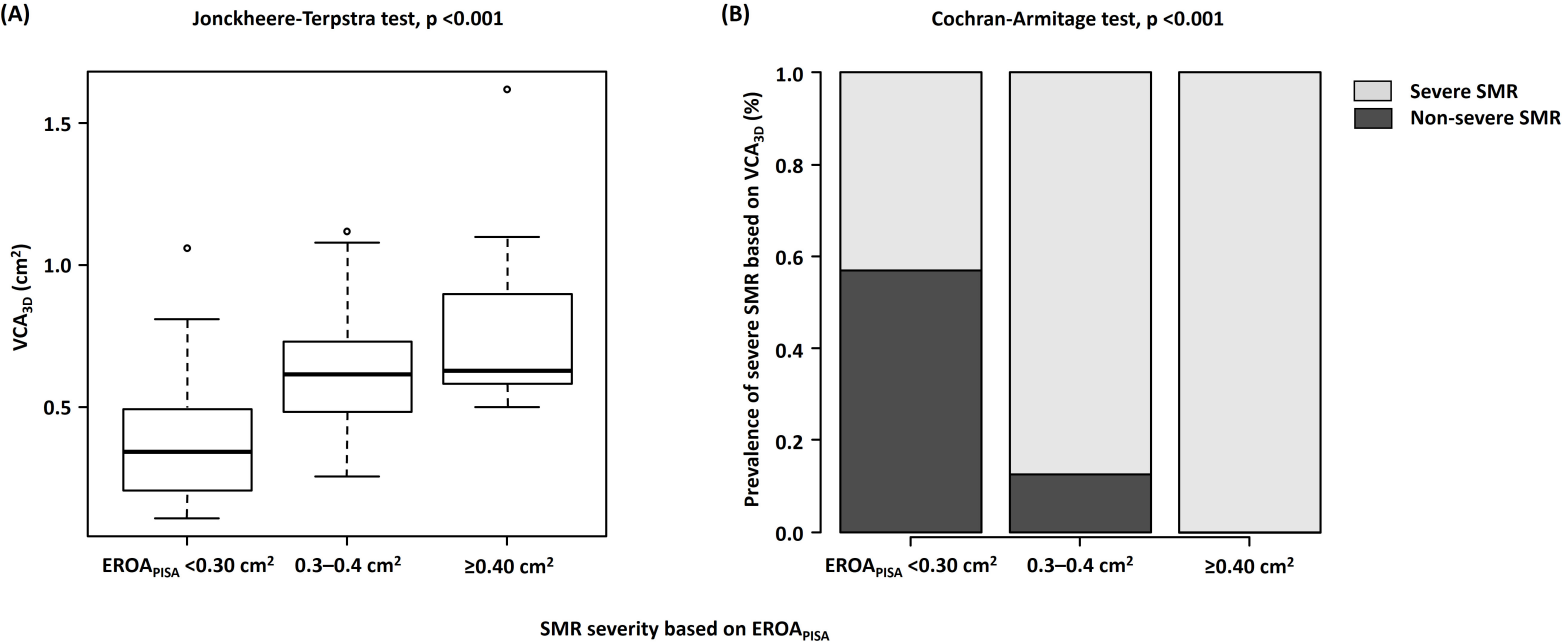
**Associations between ${\mathrm{VCA}}_{\text{𝟑𝐃}}$ and ${\mathrm{EROA}}_{\text{𝐏𝐈𝐒𝐀}}$ among the three subgroups (${\mathrm{EROA}}_{\text{𝐏𝐈𝐒𝐀}}$ of <0.30 ${\mathrm{cm}}^{2}$, ${\mathrm{EROA}}_{\text{𝐏𝐈𝐒𝐀}}$ of 0.30–0.40 ${\mathrm{cm}}^{2}$, and ${\mathrm{EROA}}_{\text{𝐏𝐈𝐒𝐀}}$ of ≥0.40 ${\mathrm{cm}}^{2}$)**. (A) Increase in ${\mathrm{VCA}}_{\text{3D}}$ according to the increase in SMR severity. (B) Incidence of severe SMR based on ${\mathrm{VCA}}_{\text{3D}}$ of ≥0.39 ${\mathrm{cm}}^{2}$ according to the increase in SMR severity. ${\mathrm{VCA}}_{\text{3D}}$, three-dimensional vena contracta area; ${\mathrm{EROA}}_{\text{PISA}}$, effective regurgitant orifice area by proximal isovelocity surface area method; SMR, secondary mitral regurgitation.

**Fig. 6.**
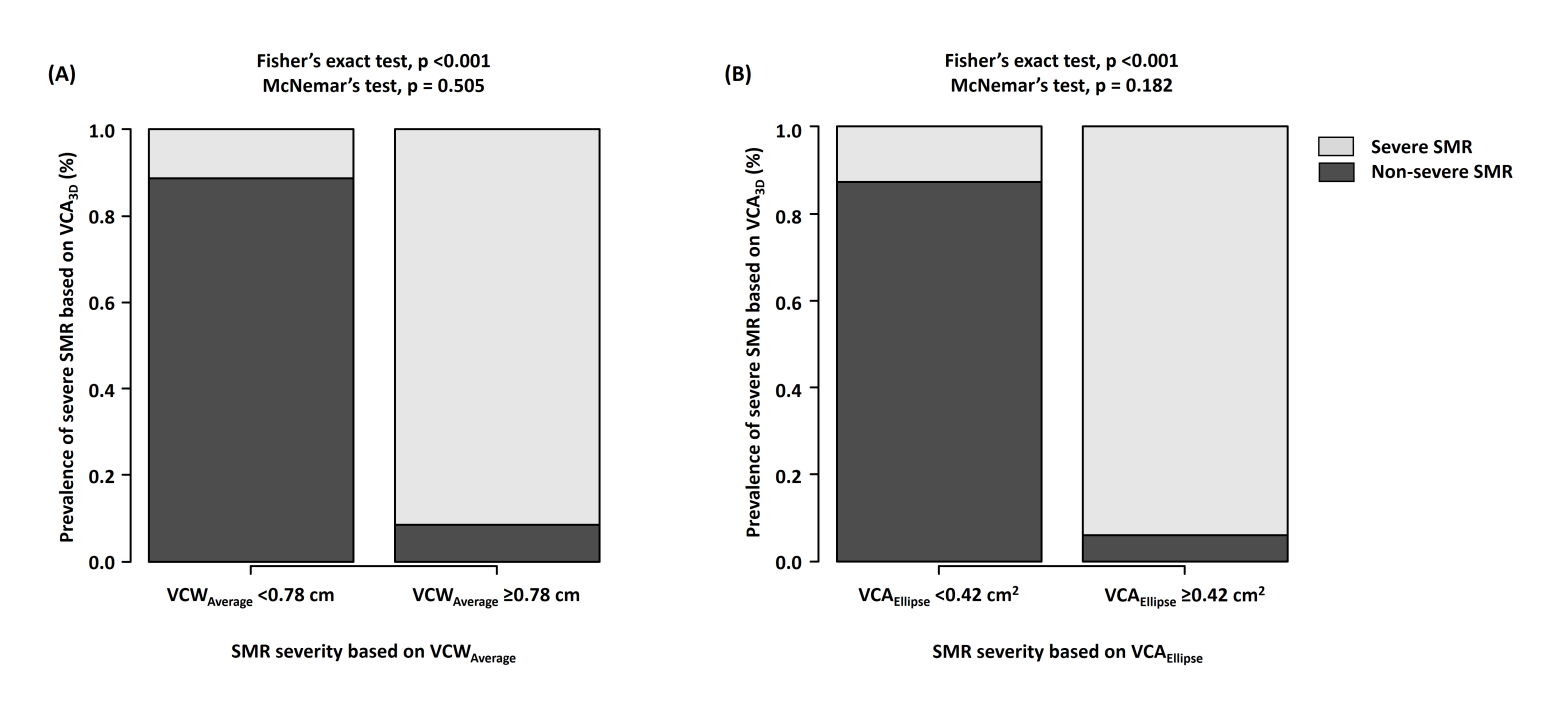
**Associations of ${\mathrm{VCA}}_{\text{𝟑𝐃}}$ with ${\mathrm{VCW}}_{\text{𝐀𝐯𝐞𝐫𝐚𝐠𝐞}}$ and ${\mathrm{VCA}}_{\text{𝐄𝐥𝐥𝐢𝐩𝐬𝐞}}$ in the ${\mathrm{EROA}}_{\text{𝐏𝐈𝐒𝐀}}$ <0.30 ${\mathrm{cm}}^{2}$ group**. (A) Incidence of severe SMR based on ${\mathrm{VCA}}_{\text{3D}}$ of ≥0.39 ${\mathrm{cm}}^{2}$ between the nonsevere (${\mathrm{VCW}}_{\text{Average}}$ of <0.78 cm) and severe (${\mathrm{VCW}}_{\text{Average}}$ of ≥0.78 cm) SMR groups. (B) Incidence of severe SMR based on ${\mathrm{VCA}}_{\text{3D}}$ of ≥0.39 ${\mathrm{cm}}^{2}$ between the nonsevere (${\mathrm{VCA}}_{\text{Ellipse}}$ of <0.42 ${\mathrm{cm}}^{2}$) and severe (${\mathrm{VCA}}_{\text{Ellipse}}$ of ≥0.42 ${\mathrm{cm}}^{2}$) SMR groups. ${\mathrm{VCA}}_{\text{3D}}$, three-dimensional vena contracta area; ${\mathrm{VCW}}_{\text{Average}}$, average of anteroposterior and mediolateral vena contracta widths; ${\mathrm{VCA}}_{\text{Ellipse}}$, vena contracta area as an ellipse; ${\mathrm{EROA}}_{\text{PISA}}$, effective regurgitant orifice area determined by the proximal isovelocity surface area method; SMR, secondary mitral regurgitation.

Our patients were classified into the following three subgroups based on ${\mathrm{EROA}}_{\text{PISA}}$ according to the current guidelines [4]: 88 patients with ${\mathrm{EROA}}_{\text{PISA}}$ of <0.30 ${\mathrm{cm}}^{2}$, 24 patients with ${\mathrm{EROA}}_{\text{PISA}}$ of 0.30–0.40 ${\mathrm{cm}}^{2}$, and 16 patients with ${\mathrm{EROA}}_{\text{PISA}}$ of ≥0.40 ${\mathrm{cm}}^{2}$. According to the incremental ${\mathrm{EROA}}_{\text{PISA}}$, ${\mathrm{VCA}}_{\text{3D}}$ (*p* < 0.001) and SMR incidence based on ${\mathrm{VCA}}_{\text{3D}}$ (*p* < 0.001) significantly increased (Fig. 5A,B). Notably, in patients with ${\mathrm{EROA}}_{\text{PISA}}$ of <0.30 ${\mathrm{cm}}^{2}$, which is suggestive of moderate SMR according to the current guidelines, 38 of 88 (43.2%) patients had severe MR based on ${\mathrm{VCA}}_{\text{3D}}$. However, SMR severity based on ${\mathrm{VCA}}_{\text{3D}}$ in patients with ${\mathrm{EROA}}_{\text{PISA}}$ of <0.30 ${\mathrm{cm}}^{2}$ was correctly reclassified as severe MR based on ${\mathrm{VCW}}_{\text{Average}}$ (*p* = 0.505) and ${\mathrm{VCA}}_{\text{Ellipse}}$ (*p* = 0.182) (Fig. 6A,B).

## 4. Discussion

The current study revealed the following findings: (1) ${\mathrm{VCW}}_{\text{Average}}$ and ${\mathrm{VCA}}_{\text{Ellipse}}$ had a fairly strong correlation with ${\mathrm{VCA}}_{\text{3D}}$, with the best cutoff values of 0.78 cm and 0.42 ${\mathrm{cm}}^{2}$, respectively, and (2) ${\mathrm{VCW}}_{\text{Average}}$ of ≥0.78 cm and ${\mathrm{VCA}}_{\text{Ellipse}}$ of ≥0.42 ${\mathrm{cm}}^{2}$ might be useful in identifying severe SMR based on ${\mathrm{VCA}}_{\text{3D}}$, particularly in patients with ${\mathrm{EROA}}_{\text{PISA}}$ of <0.30 ${\mathrm{cm}}^{2}$, corresponding to moderate SMR according to the current guidelines, who are at potential risk of underestimation of SMR severity because of the ellipticity of regurgitant orifice area [4].

### 4.1 Usefulness of ${\mathrm{VCW}}_{\text{Average}}$ and ${\mathrm{VCA}}_{\text{Ellipse}}$ in Identifying Severe SMR

Although ${\mathrm{VCW}}_{\text{AP}}$ was shown to be a reliable semiquantitative parameter for evaluating SMR severity according to the current guidelines, ${\mathrm{VCW}}_{\text{ML}}$ evaluation is not routinely used as a stand-alone parameter [4]. However, according to a previous study by Kahlert *et al*. [8], ${\mathrm{VCW}}_{\text{ML}}$ was more strongly correlated with ${\mathrm{VCA}}_{\text{3D}}$ than with ${\mathrm{VCW}}_{\text{AP}}$. Furthermore, ${\mathrm{VCW}}_{\text{Average}}$ is strongly correlated with ${\mathrm{VCA}}_{\text{3D}}$ [8]. To accurately identify severe SMR, the current guidelines recommend calculating ${\mathrm{VCW}}_{\text{Average}}$ with a cutoff value of 0.80 cm for severe SMR if the regurgitant orifice area is elliptical [4]. However, there is little information on the discrimination and best cutoff value of ${\mathrm{VCW}}_{\text{Average}}$ for severe SMR. Our study indicated that ${\mathrm{VCW}}_{\text{Average}}$ had a fairly strong correlation with ${\mathrm{VCA}}_{\text{3D}}$ and showed adequately good discrimination of severe SMR. Notably, the best cutoff value of ${\mathrm{VCW}}_{\text{Average}}$ was 0.78 cm—which is close to the value of 0.80 cm according to the current guidelines—with adequately high sensitivity and specificity for severe SMR based on ${\mathrm{VCA}}_{\text{3D}}$ [4]. Further, ${\mathrm{VCA}}_{\text{Ellipse}}$ had a strong correlation with ${\mathrm{VCA}}_{\text{3D}}$ and showed good discrimination of severe SMR. Moreover, the best cutoff value of ${\mathrm{VCA}}_{\text{Ellipse}}$ was 0.42 ${\mathrm{cm}}^{2}$, with high sensitivity and specificity for severe SMR based on ${\mathrm{VCA}}_{\text{3D}}$.

The current study and previous studies have demonstrated that the regurgitant orifice area in SMR may be elliptical [8, 12], indicating that SMR severity based on ${\mathrm{VCW}}_{\text{AP}}$ and ${\mathrm{EROA}}_{\text{PISA}}$ is underestimated [4, 5, 6]. Furthermore, there was a weak correlation between the ${\mathrm{VCA}}_{\text{3D}}$ shape index and difference between ${\mathrm{VCA}}_{\text{3D}}$ and ${\mathrm{EROA}}_{\text{PISA}}$; this finding conforms to that reported by Goebel *et al*. [11], suggesting that the ellipticity of the regurgitant orifice area rather than the extent of ellipticity is related to the underestimation of SMR severity based on ${\mathrm{EROA}}_{\text{PISA}}$.

### 4.2 Assessment of SMR Severity to Avoid its Underestimation

Patients with SMR having ${\mathrm{EROA}}_{\text{PISA}}$ of <0.30 ${\mathrm{cm}}^{2}$, corresponding to moderate SMR according to the current guidelines, have a potential risk of underestimation of SMR severity because of the elliptical regurgitant orifice area [4]. Of the 88 patients with ${\mathrm{EROA}}_{\text{PISA}}$ of <0.30 ${\mathrm{cm}}^{2}$ in the current study, 38 (43.2%) had severe MR based on ${\mathrm{VCA}}_{\text{3D}}$. In such cases, ${\mathrm{VCW}}_{\text{Average}}$ of ≥0.78 cm and/or ${\mathrm{VCA}}_{\text{Ellipse}}$ of ≥0.42 ${\mathrm{cm}}^{2}$ might be useful in identifying discordantly severe SMR based on ${\mathrm{VCA}}_{\text{3D}}$. If ${\mathrm{EROA}}_{\text{PISA}}$ is ≥0.30 ${\mathrm{cm}}^{2}$, SMR severity is expected to be truly severe based on ${\mathrm{VCA}}_{\text{3D}}$; however, ${\mathrm{EROA}}_{\text{PISA}}$ of <0.30 ${\mathrm{cm}}^{2}$ does not necessarily indicate nonsevere SMR based on ${\mathrm{VCA}}_{\text{3D}}$. If ${\mathrm{VCW}}_{\text{Average}}$ of ≥0.78 cm and/or ${\mathrm{VCA}}_{\text{Ellipse}}$ of ≥0.42 ${\mathrm{cm}}^{2}$ are calculated using ${\mathrm{VCW}}_{\text{AP}}$ and ${\mathrm{VCW}}_{\text{ML}}$, SMR severity might be considered discordantly severe despite the ${\mathrm{EROA}}_{\text{PISA}}$ of <0.30 ${\mathrm{cm}}^{2}$. After the exclusion of severe SMR according to the abovementioned assessment, symptomatic patients may be evaluated using exercise-stress echocardiography to confirm significantly worsening SMR, if applicable.

### 4.3 Clinical Implications

Although severe SMR is associated with adverse clinical outcomes [1, 2, 3], it may be underestimated using conventional echocardiographic parameters, including ${\mathrm{VCW}}_{\text{AP}}$ and ${\mathrm{EROA}}_{\text{PISA}}$. Moreover, an inaccurate assessment of SMR severity can lead to misleading indications for optimal MV interventions, including MV transcatheter edge-to-edge repair, which is known to be effective and is recommended in patients with SMR with reduced LVEF [5, 20, 21]. Karam *et al*. [22] reported that MV transcatheter edge-to-edge repair for SMR is equally effective in patients with ${\mathrm{EROA}}_{\text{PISA}}$ of <0.30 ${\mathrm{cm}}^{2}$ and those with ${\mathrm{EROA}}_{\text{PISA}}$ of ≥0.30 ${\mathrm{cm}}^{2}$ in terms of clinical outcomes, suggesting that patients with ${\mathrm{EROA}}_{\text{PISA}}$ of <0.30 ${\mathrm{cm}}^{2}$ may have a higher severity of SMR than expected based on ${\mathrm{EROA}}_{\text{PISA}}$. To obtain an accurate evaluation of SMR severity, ${\mathrm{VCA}}_{\text{3D}}$ is useful as a substantially reliable echocardiographic parameter [11]. However, the assessment of ${\mathrm{VCA}}_{\text{3D}}$ is relatively time-consuming and requires good quality of 3D-echocardiographic data [4]. ${\mathrm{VCW}}_{\text{Average}}$ and ${\mathrm{VCA}}_{\text{Ellipse}}$, which were calculated via simple equations using ${\mathrm{VCW}}_{\text{AP}}$ and ${\mathrm{VCW}}_{\text{ML}}$, showed fairly strong correlations with ${\mathrm{VCA}}_{\text{3D}}$ and good discrimination of severe SMR based on ${\mathrm{VCA}}_{\text{3D}}$. Therefore, instead of ${\mathrm{VCA}}_{\text{3D}}$, ${\mathrm{VCW}}_{\text{Average}}$ and ${\mathrm{VCA}}_{\text{Ellipse}}$, with best cutoff values of 0.78 cm and 0.42 ${\mathrm{cm}}^{2}$, respectively, might be helpful in identifying true severe SMR.

## 5. Study Limitations

This study has several important limitations. First, this was a small-scale retrospective analysis of patients with SMR who underwent TEE, with a considerable bias in data accumulation (i.e., selection bias). Second, our study defined severe SMR as ${\mathrm{VCA}}_{\text{3D}}$ of ≥0.39 ${\mathrm{cm}}^{2}$ based on the findings of a previous study [11]. However, our results may not be accurate when using other definitions of severe SMR based on modalities other than echocardiography, including cardiac magnetic resonance imaging. Third, TEE and TTE were not performed on the same day. Hence, there might have been differences in the hemodynamic status at the time of TEE and TTE. Finally, we measured ${\mathrm{VCW}}_{\text{AP}}$ and ${\mathrm{VCW}}_{\text{ML}}$ using 3D-TEE data, which may not be similar to ${\mathrm{VCW}}_{\text{AP}}$ and ${\mathrm{VCW}}_{\text{ML}}$ determined using 2D-TEE. However, there were no significant differences between ${\mathrm{VCW}}_{\text{AP}}$ and ${\mathrm{VCW}}_{\text{ML}}$ measured using 3D-TEE and 2D-echocardiography according to a previous study [8].

## 6. Conclusions

${\mathrm{VCW}}_{\text{Average}}$ and ${\mathrm{VCA}}_{\text{Ellipse}}$ based on 3D-TEE were strongly associated with ${\mathrm{VCA}}_{\text{3D}}$. Therefore, in general, the regurgitant orifice area of SMR may be elliptical, and SMR severity might be underestimated if determined using only ${\mathrm{VCW}}_{\text{AP}}$ and ${\mathrm{EROA}}_{\text{PISA}}$. Hence, ${\mathrm{VCW}}_{\text{Average}}$ and ${\mathrm{VCA}}_{\text{Ellipse}}$, with best cutoff values of 0.78 cm and 0.42 ${\mathrm{cm}}^{2}$, respectively, were useful in identifying severe SMR.

## Data Availability

Data will be shared on request to the corresponding author with the permission of New Tokyo Hospital and St. Marianna University Hospital.

## Abbreviations

SMR, Secondary mitral regurgitation; EROA, Effective regurgitant orifice area; ${\mathrm{EROA}}_{\text{PISA}}$, Effective regurgitant orifice area by proximal isovelocity surface area method; 3D-TEE, Three-dimensional transesophageal echocardiography; VC, Vena contracta; VCW, Vena contracta width; VCA, Vena contracta area; ${\mathrm{VCA}}_{\text{3D}}$, three-dimensional vena contracta area; ${\mathrm{VCA}}_{\text{Ellipse}}$, Vena contracta area as an ellipse; ${\mathrm{VCW}}_{\text{AP}}$, Anteroposterior vena contracta width; ${\mathrm{VCW}}_{\text{ML}}$, Mediolateral vena contracta width; ${\mathrm{VCW}}_{\text{Average}}$, Average of anteroposterior and mediolateral vena contracta widths.
